# Can HIV service data be used for surveillance purposes?: a case study in Guangzhou, China

**DOI:** 10.1186/s12889-018-6128-8

**Published:** 2018-11-19

**Authors:** Weibin Cheng, Huifang Xu, Fei Zhong, Stephen Pan, Joseph D. Tucker, Sharon Weir, Jinkou Zhao, Weiming Tang

**Affiliations:** 1 Dermatology Hospital of Southern Medical University, Guangzhou, China; 2 Department of AIDS Control and Prevention, Guangzhou Center for Disease Control and Prevention, Guangzhou, China; 3 University of North Carolina Project-China, No. 2 Lujing Road, Guangzhou, China; 4 School of Medicine of University of North Carolina at Chapel Hill, Chapel Hill, NC USA; 5 Technical Advice and Partnership Department, The Global Fund to Fight AIDS, Tuberculosis and Malaria, Chemin de Blandonnet 8, CH 1214 Geneva, Switzerland

**Keywords:** HIV, Sentinel surveillance, Testing service, Trend monitoring, Programmatic data, Homosexual men

## Abstract

**Background:**

Timely monitoring HIV epidemic among key populations is a formidable challenge. This study aimed to evaluate the agreement between data collected from an enhanced HIV sentinel surveillance (HSS+) and an HIV service, and to discuss whether testing service data can be used for surveillance purposes.

**Methods:**

The HSS+ data were collected from HIV sentinel surveillance conducted annually among men who have sex with men (MSM) between 2009 and 2013 in Guangzhou, China. The HIV service data were obtained from the China-Bill & Melinda Gates Foundation Cooperation Program on HIV Prevention and Care (China-Gates HIV Program) in Guangzhou during the same period. The China-Gates HIV Program aimed to increase HIV counseling and testing among MSM. We compared demographic characteristics, condom use, HIV testing history, and the HIV status among individuals in these two datasets. The Armitage-trend test was used to evaluate the HIV epidemic and behaviors of the participants in the two datasets over the study period.

**Results:**

Overall, a total of 2224 and 5311 MSM were included in the surveillance and service datasets, respectively. The majority of participants in the two datasets were between 20 and 29 years old, at least attended college, and had never been married. However, socio-demographic characteristics varied slightly between the two datasets. Similar trends were observed for the HIV epidemic in these two datasets. The surveillance dataset indicated that HIV prevalence increased from 3.9% in 2009 to 11.4% in 2013 (*P*-value for trend < 0.001), while data from the HIV service dataset indicated that MSM HIV prevalence during this same period increased from 6.2 to 8.9% (*P*-value for trend = 0.025). The rates of condom use were similar between the two datasets and remained consistent throughout the study period.

**Conclusion:**

HIV service data can complement existing HIV surveillance systems for MSM in China, though it may underestimate the HIV prevalence (HSS+ data contains people whose status is already know, while service data contains people who were initially negative or people of unknown status). HIV service data can be used for surveillance purposes, when prerequisite variables are collected from a large number people, if the quality assessment is conducted.

## Background

In China, HIV epidemic is now dominantly concentrated among men who have sex with men (MSM), and who constituted almost one-third of all cases of new HIV infections [1]. Likewise, HIV prevalence among MSM increased from 0.9% in 2003 to 8% in 2015 [2]. However, accurately monitoring infectious disease epidemics is challenge and expensive, although it is a public health priority. [3] HIV surveillance and surveys are the major data sources for measuring HIV prevalence, risk factors, intervention coverage, and the potential public health impact. There are several types of surveillance and/or surveys, including HIV sentinel surveillance (HSS), HIV sentinel surveillance plus (HSS+, with some behavior components), integrated bio-behavioral surveys (IBBS), national population-based household surveys, and programmatic mapping and size estimation exercises. To ensure accurate monitoring, representative samples usually need to be collected for HIV surveillance. However, most of the probability sampling methods considered are limited in that they are adequate only under certain circumstances and for some groups. [4] On the contrary, probability sampling strategy is challenging among key population groups who are often hidden and difficult to reach. [5] Instead of random sampling, quasi-probability methods such as time location sampling (TLS) [6] and respondent-driven-sampling (RDS) [7], and nonprobability sampling included snowball sampling and convenience sampling are often used. [5]

As one type of HIV surveillance, HSS+ has been successfully implemented in many countries, included China, and plays an important role in tracking the trend of the HIV epidemic and behaviors among key populations. [8–10] However, strong operational capacity is usually needed for HSS+ to functionally successfully. Hence, HSS+ implementation in resource-limited settings remains a challenge. For example, due to resource constraints in the rural countryside and small cities, all HSS+ for men MSM in China are conducted exclusively in large cities. Additional methods are urgently needed for monitoring HIV epidemic and behavioral trends among key populations in limited resource settings.

HIV services are often used to identify undiagnosed cases of HIV infection. Different from HSS+ (of which, individuals already diagnosed with HIV are included), the source population of HIV service is people whose HIV status is negative or unknown before the testing (individuals already diagnosed with HIV are excluded), based on self-report. HIV services usually are not developed for biological and behavior monitoring and do not have a sampling plan. Due to its convenience, this method has been widely implemented, even in resource-constrained countries. In China, HIV services routinely collect demographic and brief behavioral information. For example, the China-Gates HIV Program aimed to increase HIV counseling and testing, to identify HIV cases and link them to care. Although the essential purpose was different from surveillance, it may be useful for monitoring HIV epidemic among MSM in low and middle-income countries (LMIC). For this reason, the Global Fund and the World Health Organization (WHO) recommended that programmatic results be used for surveillance purposes, if possible [11]. For example, the WHO issued guidelines for assessing the utility of data from prevention of mother-to-child transmission in 2013. [12]

The purposes of this study are to evaluate whether the HIV prevalence and behavior data, collected from HSS+ and HIV service among MSM in Guangzhou, China, agree with each other; to discuss how programmatic data may be used for surveillance purposes, and to propose what minimum conditions are required if HIV service data can be used for surveillance. The reason for conducting this study in Guangzhou is the HIV service data in this city were mainly collected by one community-based organization (CBO), while the same CBO collected the HSS+ data in the different period of the same year, and the quality of the two datasets is comparable.

## Methods

### Design

The HSS+ data in this study were retrieved from HIV sentinel surveillance sites maintained from 2009 to 2013 in Guangzhou, China. Moreover, HIV service data were collected from the China-Bill & Melinda Gates Foundation Cooperation Program on HIV Prevention and Care (China-Gates HIV Program) between 2009 and 2013. This testing service promoted collaboration between public sector agencies and CBOs in the delivery of prevention and support services for key populations, including MSM.

### Recruitment

#### HSS+

Guangzhou is one of China’s national HSS+ sites for monitoring MSM HIV trends and related behaviors. Serial cross-sectional HSS+ were conducted between April and July of each year between 2009 and 2013. For easy access, surveys were completed at a local CBO operated for MSM and other sexual minority groups (Guangzhou Tongzhi/ Lingnan Partner Community Support Center). This site was supported by the Guangzhou Center for Disease Control and Prevention (CDC). Eligibility criteria required that participants be male, have had anal or oral sex with men in the last 12 months, be at least 18 years old and have lived in Guangzhou for at least six months. Snowball sampling was used to recruit participants. MSM who went to the center for HIV testing services were consecutively recruited into the survey. Participants of specific characteristic constitution were served as seeds to recruit MSM of the same group. Characteristics balance was monitored during recruitment in order to keep the sample characteristics in consistent with previously RDS survey results [13]. A complimentary gift (worth about $3 USD) was provided to each participant who completed the face-to-face interview and provided 5 ml blood for serological testing. To simplify, in this reported manuscript, we will call HSS+ data as surveillance data.

The sample size for surveillance in each year was estimated based on the Chinese National HIV sentinel surveillance protocol and recommendations of the WHO. [14] The expected number of participants in each year was 400. We have described this in detail somewhere else. [15]

#### HIV service

The China-Gates HIV Program in Guangzhou was tailored to MSM and offered free HIV and syphilis testing service every three months. It eventually conducted 14,268 times of HIV tests for MSM between 2009 and 2013. To make the two data sets independent (some participants in the HIV service were also included in the surveillance during the surveillance period), and ensure one MSM was only caught once in each year, we only used HIV service data that were collected between September and December of each study year.

Appendix used one formula we developed to reflect the differences between the HIV prevalence monitored by urveillance and HIV-positive rate identified from testing service data. From the formula, the difference between the two proportions can be represented as N^^^*B/[(N^^^+A + B)*(N^^^+A)]. N^^^ represents the total number of MSM whose HIV serostatus is negative at a given time point, A represents the total number of people whose HIV serostatus is positive, but not identified at the same time point, while B represents the total number of people whose HIV serostatus is positive and identified at this time point. Here, N refers to the total number of MSM in a given city and N = N^^^+A + B. This difference indicated a dynamic correlation between HIV prevalence monitored by surveillance and HIV-positive rate in HIV service.

### Demographic and behavioral measures

During each year of the surveillance, socio-demographic and behavioral information was collected through a face-to-face structured interview. Detailed information about study measures has been described elsewhere. [16] Briefly, socio-demographic information such as age, marital status, and educational attainment was collected. Behavioral measures included: frequency of condom use in the last six months and condom use during last anal sex with a male partner. Each participant also reported the number of male sexual partners in the last six months and information on HIV testing history.

Different from surveillance, the variables of interest that were collected from testing services were a slightly different year by year. The following sociodemographic and behavioral variables were collected from HIV service: age, marital status, residence, profession, educational attainment, and condom use during anal intercourse with male and number of male sexual partners in the past three months.

In this study, inconsistent condom use was defined as any condomless anal intercourse with a male within the specific period (i.e., three or six months).

According to Appendix, the HIV prevalence measured in surveillance was calculated as HIV prevalence = (A + B)/(N′ + A + B), where A + B are all the HIV positive cases identified in surveillance, and N′ + A + B represents all the recruited MSM in surveillance. In HIV service, HIV positive rates = A/(N′ + A). As we mentioned in Appendix represents the total number of newly identified HIV cases in HIV service, and N′ + A represents all the recruited participants whose HIV status is negative or unknown before the testing.

### Serological testing

Surveillance and testing service used the same serological testing algorithm. In this serological testing algorithm, five ml of venous blood was collected from each participant for HIV antibody testing using the standard protocol and laboratory methods of China National Center for STD and AIDS Control. From 2009 to 2011, HIV screening was done by using enzyme-linked immunoassays (ELISAs; Diagnostic Kit for Antibody to Human Immunodeficiency Virus, BioMérieux, Boxtel, The Netherlands, Beijing BGI-GBI Biotech, Beijing, China). In 2012 to 2013, HIV screening was done by using HIV rapid test kit (Colloidal Gold; Diagnostic Kit for Antibody to Human Immunodeficiency Virus, InTec PRODUCTS, INC., Xiamen, China). Any HIV-positive test by ELISA method or rapid test was referred for confirmatory testing by using Western Blot (WB; MP Biomedicals Asia Pacific Pte Ltd., Singapore). All tests were conducted under the supervision of the Guangzhou CDC HIV confirmatory laboratory. Anonymized test results were linked with interview data using the UIDs. For surveillance, HIV prevalence was calculated by using the number of HIV positive cases divided by total sample survey in each year. For testing service, HIV positive rate was substituted for HIV prevalence.

Both surveillance and the HIV service offered each participant HIV risk-reduction counseling and pre- and post-test counseling services. Participants confirmed as HIV positive were referred to the Chinese HIV/AIDS Care system, and was linked to care.

### Ethics statement

Written informed consent was obtained from each participant prior to the survey for both surveillance and the testing service. The study was carried out in accordance with approved guidelines. The ethics review committee of Guangzhou CDC reviewed and approved the study protocol.

### Data analysis

Surveillance data were double entered and cleaned using EpiData (version 3.1, Denmark). HIV service data were double entered and cleaned using Microsoft Excel 2003.

Statistical analysis was performed using SAS 9.3 (Cary, NC, US). Descriptive analysis was conducted to explore the demographic and behavioral variables in the two datasets. Chi-square tests were used to examine differences between surveillance and the HIV service during the study period. The Armitage-trend test was used to test for significant trends in HIV prevalence and behaviors between 2009 and 2013.

## Results

Between 2009 and 2013, a total of 2224 MSM were recruited into the surveillance, and 5311 MSM (conducted between September and December of each year) were included in the testing service. In both datasets, the majority of participants were between 20 and 29 years old, had at least college-level educational attainment, and had never married. However, sociodemographic characteristics varied slightly between the two datasets. For example, the participants in the testing service sample were younger and more likely to be unmarried. (Table 1).

**Table 1:** Sociodemographic Characteristics of MSM recruited in different programs in Guangzhou, China between 2009 and 2013 (*N* = 7535)

Variables	Testing services (*n* = 5311)		Surveillance (*n* = 2224)	
	Frequency	Percent	Frequency	Percent
Age				
< 20	221	4.2	59	2.6
20–29	3306	62.2	1319	59.3
30–39	1370	25.8	647	29.1
40 or above	410	7.7	199	9.0
Missing	4	0.1	–	–
Education				
Junior high school or below	1247	23.5	817	36.7
College or above	3382	63.7	1407	63.3
Missing	682	12.8	–	–
Marital Status				
Single	3705	69.8	1436	64.6
Cohabitating	216	4.1	344	15.5
Married	566	10.7	377	17.0
divorced	111	2.1	67	3.0
Missing	713	13.4	–	–

**Fig. 1:**
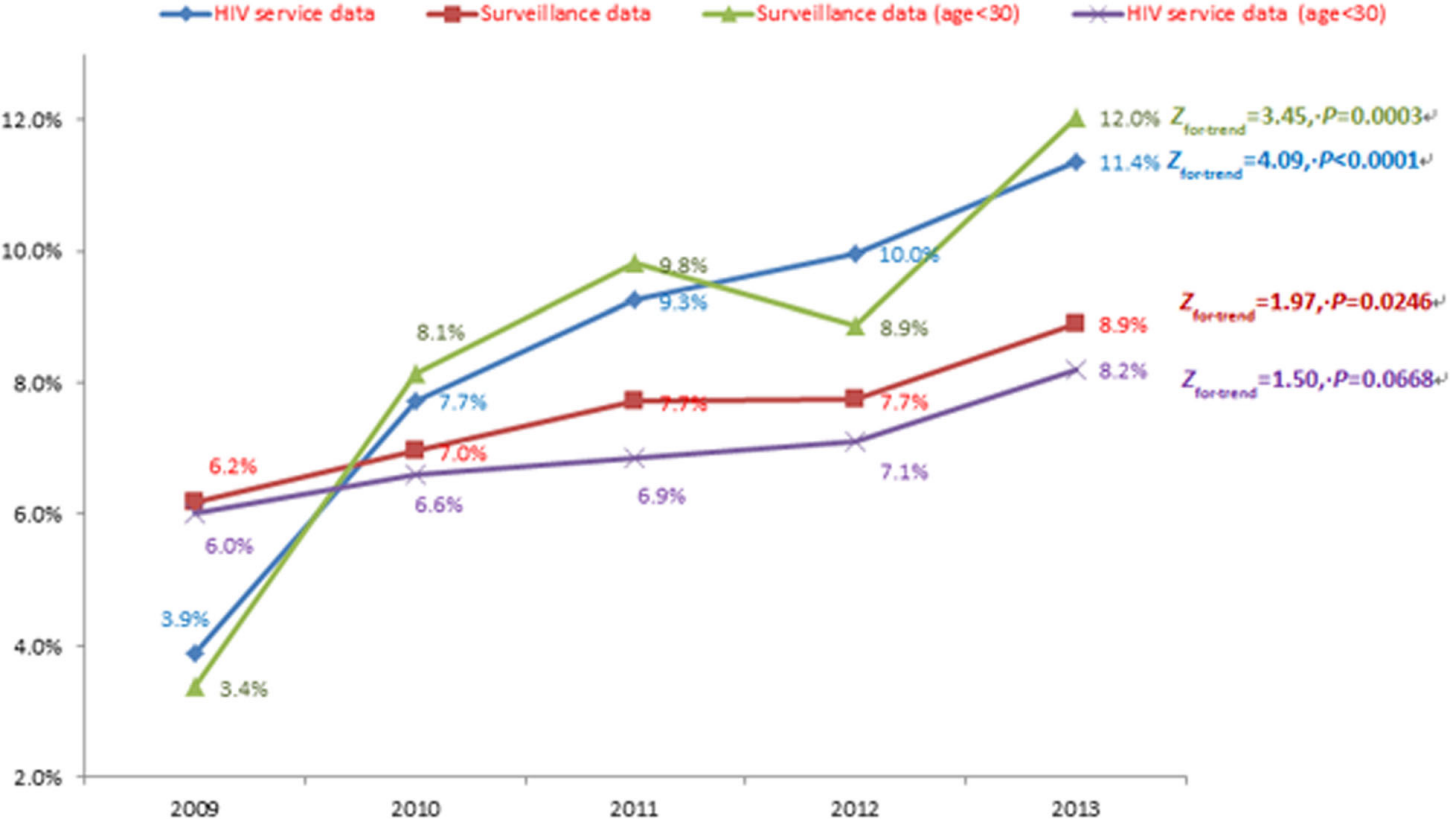
The trend of HIV epidemic among MSM recruited in different programs between 2009 and 2013 in Guangzhou, China (N = 7535)

Figure 1 indicates that HIV prevalence increased during the study period in both datasets. The surveillance reported that HIV prevalence rose from 3.9% in 2009 to 11.4% in 2013 (*Z*_for trend_ = 4.09, *P* < 0.001), and data from the HIV service indicated that HIV-positive rate during this same time period increased from 6.2 to 8.9% (*Z*_for trend_ = 1.97, *P* = 0.025). In addition, the difference between HIV prevalence and HIV positive rate were increased over time. Similar results were found for young participants (< 30 years old).

Among participants who attend surveillance, 31.3% had never tested for HIV before, and 30.7% had tested in the past six months. In addition, 59.7% reported more than one male partner in the past six months, and 57.8% had engaged in condomless anal intercourse with a male partner in the past six months. (Table 2).

**Table 2:** Testing and sexual behaviors of MSM recruited in different programs in Guangzhou, China between 2009 and 2013 (N = 7535)

Variables	Testing service^a^(*n* = 5311)		Surveillance (*n* = 2224)	
	Number	Frequency	Number	Frequency
HIV testing record				
*Never tested*	*1317*	*28.0*	*696*	*31.3*
*Tested in the past 6 months*	*1051*	*22.3*	*683*	*30.7*
*Ever tested*	*2339*	*49.7*	*845*	*38.0*
No. of male partners in the last 3 months				
*0*	*302*	*10.0*	*N/A*	*N/A*
*1*	*1512*	*50.0*	*N/A*	*N/A*
*2 to 3*	*1016*	*33.6*	*N/A*	*N/A*
*4 to 9*	*158*	*5.2*	*N/A*	*N/A*
*10 or more*	*36.0*	*1.2*	*N/A*	*N/A*
No. of male partners in the last six months				
*0*	*N/A*	*N/A*	*210*	*9.4*
*1*	*N/A*	*N/A*	*687*	*30.9*
*2 to 3*	*N/A*	*N/A*	*921*	*41.5*
*4 to 9*	*N/A*	*N/A*	*311*	*14.0*
*10 or more*	*N/A*	*N/A*	*93*	*4.2*
Condomless anal intercourse with male in the last three months				
*Yes*	*1099*	*37.1*	*N/A*	*N/A*
*No*	*2963*	*62.9*	*N/A*	*N/A*
Condomless anal intercourse with male in the last six months				
*Yes*	*N/A*	*N/A*	*1286*	*57.8*
*No*	*N/A*	*N/A*	*938*	*42.2*
Used condom during the last intercourse with male				
*Yes*	*1945*	*69.0*	*1383*	*68.7*
*No*	*873*	*31.0*	*630*	*31.3*

^a^For HIV service data, 604 cases were not known their HIV testing records were excluded from analysis. The “Ever tested” in HIV service data presented the sample of the date of the last test was not available. For the change of the program data collection plan in 2013, No. of male partners in the last three months, condomless anal intercourse with a male in the last three months, and used a condom during the last intercourse with male information were not available

Among MSM who utilized the HIV service, 28.0% had never tested for HIV before, but only 22.3% tested for HIV in the past six months. In addition, 40.0% of MSM in the HIV service reported that they had more than one male partner in the past three months, and 37.1% had engaged in condomless anal intercourse with a male partner in the past three months. (Table 2).

**Fig. 2:**
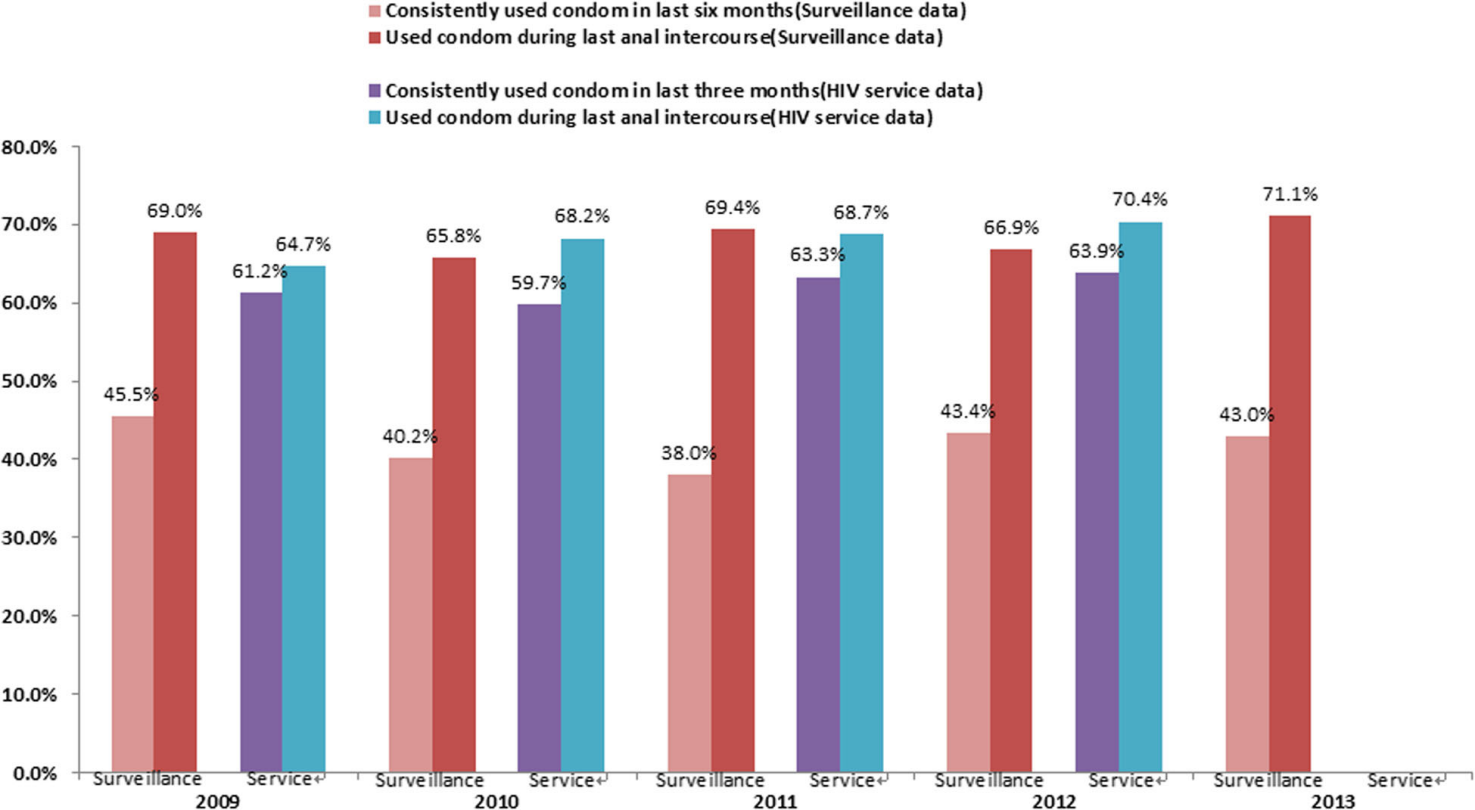
Sexual behaviors of MSM recruited in different programs in Guangzhou, China between 2009 and 2013 (*N* = 7535)

Prevalence of inconsistent condom use during anal intercourse was remained steady and high throughout the study period, ranging from 54.5 to 62% in the past six months in surveillance (*Z*_for trend_ = 0.06, *P* = 0.48) and 36.1% to 40.3% in the past three months in HIV service (*Z*_for trend_ = 1.44, *P* = 0.076). Condom use at last anal intercourse was stable and comparable between the two datasets throughout the observed study period, ranging from 65.8 to 71.1% in surveillance (*Z*_for trend_ = 0.96, *P* = 0.17) and 64.7% to 70.4% in the HIV service (*Z*_for trend_ = 1.62, *P* = 0.052). (Figure2).

## Discussion

Timely monitoring of the HIV epidemic is essential for planning appropriate public health responses. [17] Both HIV surveillance and programmatic data indicated that the burden of HIV among MSM in Guangzhou is spreading, and a large proportion of MSM engage in condomless sex. Our study extends the existing literature by comparing HIV surveillance and service data, evaluating whether programmatic data is suitable for surveillance purposes, and discussing minimum requirements when using testing data for surveillance purpose. Study results suggest that HIV service can be used for HIV surveillance, though it may under estimate the HIV prevalence.

Both HIV surveillance and programmatic data showed that many participants engaged in condomless sex, and the proportions of MSM used a condom at last anal sex in the two datasets are similar. These findings are consistent with the overall data collected through Chinese HSS+ during 2009 and 2013. [15] Though the source populations varied slightly between these two datasets, there are two reasons why condom use in the last anal sex may have been comparable. First, the only difference regarding the source population for these two datasets is that people diagnosed with HIV were excluded from the testing service, and this group of MSM is relatively small. For example, in China, the overall reported HIV prevalence among MSM was 8.0% in 2015 [18], and only about 50% of people living with HIV were identified [19]. Hence, only 4% of the target population may have differed between the two data sets. Second, people living with HIV in China tend to have similar condom use patterns as HIV-negative MSM, or whose status was unknown. [20]

Both the surveillance and HIV service data also indicated a rising trend of HIV among MSM in Guangzhou, China. This trend is consistent with national trends among MSM. Between 2009 and 2015, HIV prevalence among MSM nationwide increased from 5.5 to 8.0%. [15, 18] However, the HIV prevalence in Guangzhou is higher than majority of other cities of China [15], but it is lower than the HIV prevalence among MSM in the US. [21, 22] Overall, HIV prevalence in the surveillance was slightly higher than the positive rate of the programmatic data, except the year of 2009 [23]. This difference in HIV prevalence between the two datasets is likely because surveillance included known positive cases that testing service did not. The primary purpose of surveillance is to monitor HIV prevalence among MSM specifically. However, the testing service aims to identify undiagnosed cases of HIV. Thus individuals already diagnosed with HIV are excluded from testing services. Theoretically, HIV prevalence in surveillance should be higher than or equal to the proportion of people who test positive in the testing services, which was shown in the formula developed by our study team (Appendix).

However, when using testing service data for surveillance purpose, several things need to be taken into consideration. First, before used for surveillance purpose, a quality assessment of the data collected from the testing system should be conducted, to evaluate the magnitude and direction of selection bias as well as other bias. [24] Second, the sample size of testing service data should be sufficient to assess the epidemic (should be similar to or large than the sample size requirement of surveillance, for example, 400), the testing service data should be collected in the same period of each year (in order to prevent the sample persons being caught for multiple times in the same year), and last for a certain years (I.e, 3 or more years, in order to get the continuous measure for trend analysis). Third, for the site selection, testing service data should be only used in places that data from HIV surveillance cannot provide the information needed for surveillance (i.e., place without HIV sentinel surveillance).(9) Fourth, based on the comparisons of these two datasets, in order to adjust the prevalence estimation by using testing service data, the minimum set of variables should be collected for surveillance (I.e, age, date of visit, residence, educational level, HIV and syphilis test result, and previously known HIV-positive status). If possible, a set of key behavioral variables (condom use with male/female partner, HIV testing history and the number of partners in a certain time) should also be included. Fifth, feedback from the local community showed that, using the HIV service data is ease of implementation and more readily accept by the client, because information collected from service program was much less than the sentinel surveillance. While challenges remain when HIV service conducting in the outreach programs where testing was not applicable.

Our study has several limitations. First, due to the source population of the two datasets are different, we cannot make direct comparisons between the HIV epidemic and behavioral measures collected. However, the source populations of these two datasets are only slightly different. Second, due to the sentinel protocol, surveillance data for the year 2009 have been subject to strong selection bias because sample strategy was set to target MSM who have been recruited in the 2008 survey. This may explain why the proportion of people living with HIV in the programmatic data was higher than the prevalence measured by surveillance in 2009. Third, surveillance data were collected through snowball sampling and may have been relatively reliable when compare with the HIV service programmatic data, which were collected through convenience sampling. Due to the sampling strategies or random error, both surveillance and HIV service data are subject to bias, conducting quality assessment and estimate the trend and magnitude of the bias would be useful.

## Conclusion

We conclude that routinely collected HIV service data is an imperfect but suitable method for biological surveillance and behavior surveillance among MSM in China. Resource-limited settings without surveillance can use HIV service data to evaluate HIV epidemic trends as well as behavioral trends of key populations. However, the innate defect of service data that contains people who were initially negative or people of unknown status which may be underestimate of the HIV prevalence. HIV service data can be used for surveillance purposes when prerequisite variables are also collected in a large enough sample from corresponding sentinel sites, and quality assessment of the magnitude and direction of bias is conducted.

## Appendix

### Appendix Difference between HIV epidemic among IBBS data and HIV testing program data

Appendix presents a formula that represents the differences between the HIV prevalence monitored by HSS+ and the proportion of people who test positive in HIV test program data. From the formula, the difference between the two proportions can be represented as N^^^*B/[(N^^^+A + B)*(N^^^+A)]. N^^^ represents the total number of MSM whose HIV serostatus is positive at a given time point, A represents the total number of people whose HIV serostatus is positive, but not identified at the same time point, while B represent the total number of people whose HIV serostatus is positive and identified at this time point. While N (the total number of MSM in a given place) = N^^^+A + B. This difference indicates a correlation between HIV prevalence monitored by HSS+ and the HIV positive rate among people in the HIV testing program.

**Hypothesis:** $$ \frac{\mathrm{A}+\mathrm{B}}{{\mathrm{N}}^{\prime }+\mathrm{A}+\mathrm{B}}.>.\frac{\mathrm{A}}{{\mathrm{N}}^{\prime }+\mathrm{A}} $$

Then the difference between the two will be:

$$ D=.\frac{\mathrm{A}+\mathrm{B}}{{\mathrm{N}}^{\prime }+\mathrm{A}+\mathrm{B}}.-.\frac{\mathrm{A}}{{\mathrm{N}}^{\prime }+\mathrm{A}}. $$

$$ D=.\frac{\left(\mathrm{A}+\mathrm{B}\right)\left({\mathrm{N}}^{\prime }+\mathrm{A}\right)}{\left({\mathrm{N}}^{\prime }+\mathrm{A}+\mathrm{B}\right)\left({\mathrm{N}}^{\prime }+\mathrm{A}\right)}.-.\frac{\left({\mathrm{N}}^{\prime }+\mathrm{A}+\mathrm{B}\right)\cdotp \mathrm{A}}{\left({\mathrm{N}}^{\prime }+\mathrm{A}+\mathrm{B}\right)\left({\mathrm{N}}^{\prime }+\mathrm{A}\right)} $$

$$ D=.\frac{{\mathrm{BN}}^{\prime }}{\left({\mathrm{N}}^{\prime }+\mathrm{A}+\mathrm{B}\right)\left({\mathrm{N}}^{\prime }+\mathrm{A}\right)} $$

**Note:** D refers to the difference between HIV prevalence among MSM in HSS+ and HIV positive rate among MSM in HIV testing program.

## Abbreviations

AIDS: Acquired immune deficiency syndrome
HIV: Human immunodeficiency virus
HSS+: HIV sentinel surveillance plus
HSS: HIV sentinel surveillance
LMIC: Low and middle-income countries
RDS: Respondent-driven-sampling
STD: Sexually transmitted diseases
TLS: Time location sampling (TLS)
WHO: World Health Organization

## References

[1] Ministry of Health of the People's Republic of China. China AIDS response progress report. Beijing, UNAIDS; 2012.

[2] Ibrahim S, Al Attas SA, Mansour GA, Ouda S, Fallatah H (2015). Accuracy of rapid oral HCV diagnostic test among a Saudi sample. Clin Oral Investig.

[3] Nsubuga P, White ME, Thacker SB, Anderson MA, Blount SB, Broome CV, Chiller TM, Espitia V, Imtiaz R, Sosin D (2006). Public health surveillance: a tool for targeting and monitoring interventions. Disease control priorities in developing countries.

[4] HIV/AIDS JUNPo, Organization WH (2003). Guidelines for conducting HIV sentinel serosurveys among pregnant women and other groups. Guidelines for conducting HIV sentinel serosurveys among pregnant women and other groups.

[5] Magnani R, Sabin K, Saidel T, Heckathorn D (2005). Review of sampling hard-to-reach and hidden populations for HIV surveillance. Aids.

[6] Zhao J, Cai R, Chen L, Cai W, Yang Z, Richardus JH, de Vlas SJ (2015). A comparison between respondent-driven sampling and time-location sampling among men who have sex with men in Shenzhen, China. Arch Sex Behav.

[7] Tang W, Yang H, Mahapatra T, Huan X, Yan H, Li J, Fu G, Zhao J, Detels R (2013). Feasibility of recruiting a diverse sample of men who have sex with men: observation from Nanjing, China. PLoS One.

[8] Elhadi M, Elbadawi A, Abdelrahman S, Mohammed I, Bozicevic I, Hassan EA, Elmukhtar M, Ahmed S, Abdelraheem MS, Mubarak N. Integrated bio-behavioural HIV surveillance surveys among female sex workers in Sudan, 2011–2012. Sex Transm Infect. 2013. pp.sextrans-2013. 10.1136/sextrans-2013-051097 PMC3841728 23996450

[9] Tun W, Sheehy M, Broz D, Okal J, Muraguri N, Raymond HF, Musyoki H, Kim AA, Muthui M, Geibel S (2015). HIV and STI prevalence and injection behaviors among people who inject drugs in Nairobi: results from a 2011 bio-behavioral study using respondent-driven sampling. AIDS Behav.

[10] Ruan S, Yang H, Zhu Y, Wang M, Ma Y, Zhao J, McFarland W, Raymond HF (2009). Rising HIV prevalence among married and unmarried among men who have sex with men: Jinan, China. AIDS Behav.

[11] Billong SC, Dee J, Fokam J, Nguefack-Tsague G, Ekali GL, Fodjo R, Temgoua ES, Billong EJ, Sosso SM, Mosoko JJ (2017). Feasibility study of HIV sentinel surveillance using PMTCT data in Cameroon: from scientific success to programmatic failure. BMC Infect Dis.

[12] Organization WH (2013). Guidelines for assessing the utility of data from prevention of mother-to-child transmission (PMTCT) programmes for HIV sentinel surveillance among pregnant women.

[13] Zhong F, Lin P, Xu H, Wang Y, Wang M, He Q, Fan L, Li Y, Wen F, Liang Y (2011). Possible increase in HIV and syphilis prevalence among men who have sex with men in Guangzhou, China: results from a respondent-driven sampling survey. AIDS Behav.

[14] Lin W, Chen S, Seguy N, Chen Z, Sabin K, Calleja JG, Bulterys M (2012). Is the HIV sentinel surveillance system adequate in China? Findings from an evaluation of the national HIV sentinel surveillance system. Western Pac Surveill Response J.

[15] Qin Q, Tang W, Ge L, Li D, Mahapatra T, Wang L, Guo W, Cui Y, Sun J. Changing trend of HIV, syphilis and hepatitis C among men who have sex with men in China. Sci Rep. 2016;6:31081. 10.1038/srep31081 PMC4989164 27535092

[16] Cheng W, Tang W, Zhong F, Babu GR, Han Z, Qin F, Gao K, Mai H, Zhao Y, Liang C (2014). Consistently high unprotected anal intercourse (UAI) and factors correlated with UAI among men who have sex with men: implication of a serial cross-sectional study in Guangzhou, China. BMC Infect Dis.

[17] Pisani E, Lazzari S, Walker N, Schwartländer B (2003). HIV surveillance: a global perspective. J Acquir Immune Defic Syndr.

[18] Tang S, Tang W, Meyers K, Chan P, Chen Z, Tucker JD (2016). HIV epidemiology and responses among men who have sex with men and transgender individuals in China: a scoping review. BMC Infect Dis.

[19] Sullivan SG, Wu Z, Detels R (2010). Missed opportunities for HIV testing and counselling in Asia. Aids.

[20] Forney JC, Miller RL (2012). Risk and protective factors related to HIV-risk behavior: a comparison between HIV-positive and HIV-negative young men who have sex with men. AIDS Care.

[21] HIV PREVALENCE IN MEN WHO HAVE SEX WITH MEN.

[22] Beyrer C, Baral SD, van Griensven F, Goodreau SM, Chariyalertsak S, Wirtz AL, Brookmeyer R (2012). Global epidemiology of HIV infection in men who have sex with men. Lancet.

[23] Zhong F, Liang B, Xu H, Cheng W, Fan L, Han Z, Liang C, Gao K, Mai H, Qin F (2014). Increasing HIV and decreasing syphilis prevalence in a context of persistently high unprotected anal intercourse, six consecutive annual surveys among men who have sex with men in Guangzhou, China, 2008 to 2013. PLoS One.

[24] Plate DK (2007). Group RHTEW: Evaluation and implementation of rapid HIV tests: the experience in 11 African countries. AIDS Res Hum Retrovir.

